# Accelerated Idioventricular Rhythm at the Termination of an Episode of Vasospastic Angina

**DOI:** 10.7759/cureus.3895

**Published:** 2019-01-16

**Authors:** Mahaletwork Assefa, Senan J Yasar, Obai Abdullah, Hee Kong Fong, Sudarshan Balla

**Affiliations:** 1 Internal Medicine, University of Missouri, Columbia, USA; 2 Cardiology, University of Missouri, Columbia, USA; 3 Cardiology, West Virginia University, Morgantown, USA

**Keywords:** coronary vasospasm, printzmetal angina, accelerated idioventricular rhythm

## Abstract

A 57-year-old male with gastroesophageal reflux disease and esophageal stricture with dilation presented as a cardiac catheterization lab activation for anterolateral ST-segment elevation myocardial infarction. He had suffered an unwitnessed syncopal episode after severe substernal chest pain. Electrocardiogram (ECG) showed anterolateral ST-segment elevation. Markers of myocardial injury were negative. He subsequently had an unremarkable coronary angiogram, echocardiogram, and cardiac magnetic resonance imaging (MRI). He had another episode of crushing chest pain and palpitations during his hospital stay, which correlated with ST-segment elevation, followed by a slow run of ventricular arrhythmia that terminated after a dose of sublingual nitroglycerin. A diagnosis of accelerated idioventricular rhythm (AIVR) following coronary artery vasospasm (CAV) was made. This clinical vignette presents a unique presentation of AIVR following an episode of Prinzmetal’s angina.

## Introduction

Coronary artery vasospasm (CAV) is a constriction of coronary arteries and has been documented as a cause of myocardial injury, as well as fatal arrhythmia since the late 1950s [1]. It can cause transient, complete to near-complete occlusion of coronary vessels leading to myocardial injury. Accelerated idioventricular rhythm (AIVR), a slow transient ventricular arrhythmia, could be used as a marker of reperfusion [2]. However, its association with CAV events in the setting of normal serum troponin has not been well established.

## Case presentation

A 57-year-old Caucasian male was brought in by emergency medical services (EMS) with reports of severe substernal chest pain. The cardiac catheterization lab was activated for anterolateral ST-segment elevation noted on the electrocardiogram (ECG). He reported severe substernal chest pressure and belching followed by an unwitnessed syncopal episode. He had several seconds of cardiopulmonary resuscitation performed by bystanders prior to electromyostimulation (EMS) arrival. His past medical history includes gastroesophageal reflux disease and esophageal stricture with dilation.

**Figure 1:**
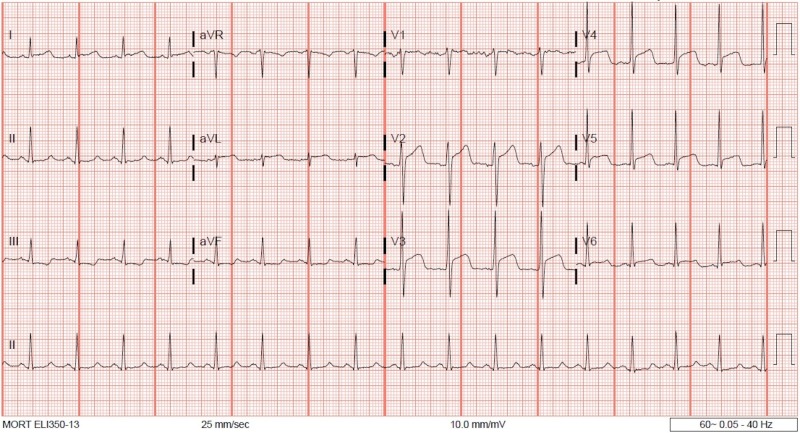
12-Lead electrocardiogram (ECG) at presentation showing ST-segment elevation in anterolateral leads. aVR, augmented vector right; aVL, augmented vector left; aVF, augmented vector foot.

**Figure 2:**
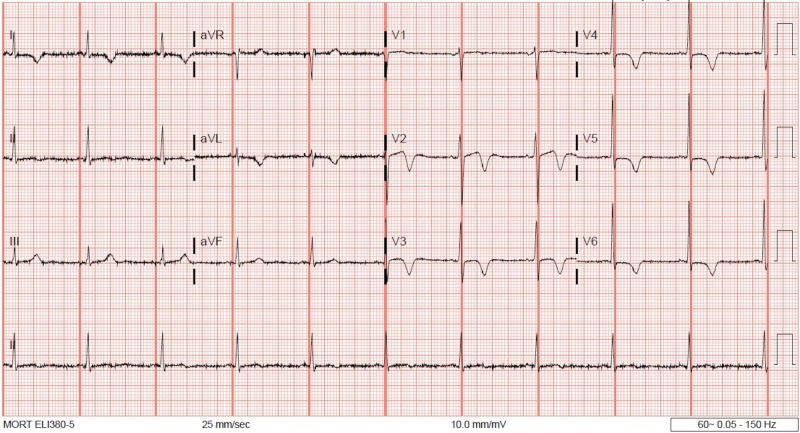
12-Lead ECG post-resolution of ST-segment elevation, with new T wave inversion in anterolateral leads. aVR, augmented vector right; aVL, augmented vector left; aVF, augmented vector foot.

**Figure 3:**
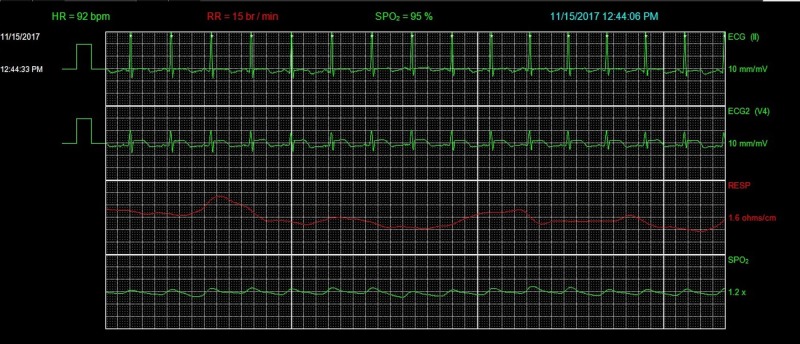
Telemetry showing ST-segment elevation in Lead V4. HR, heart rate; RR, respiratory rate; SPO_2_, peripheral capillary oxygen saturation; ECG, electrocardiogram.

**Figure 4:**
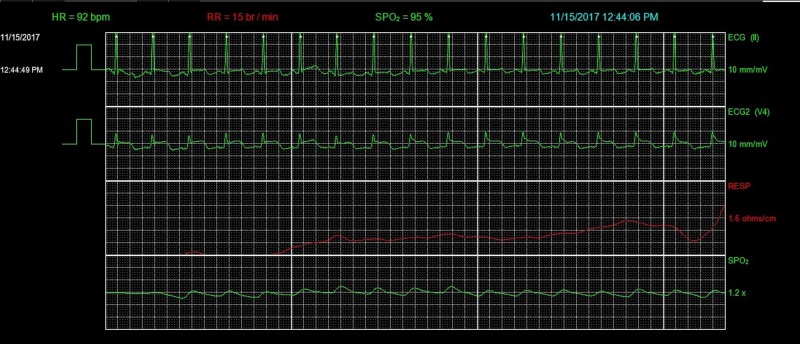
Telemetry showing worsening ST-segment elevation in Lead V4. HR, heart rate; RR, respiratory rate; SPO_2_, peripheral capillary oxygen saturation; ECG, electrocardiogram.

**Figure 5:**
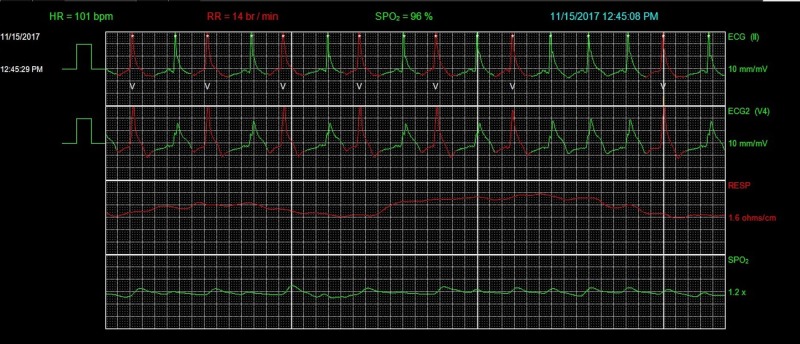
Telemetry showing accelerated idioventricular rhythm (AIVR). HR, heart rate; RR, respiratory rate; SPO_2_, peripheral capillary oxygen saturation; ECG, electrocardiogram.

**Figure 6:**
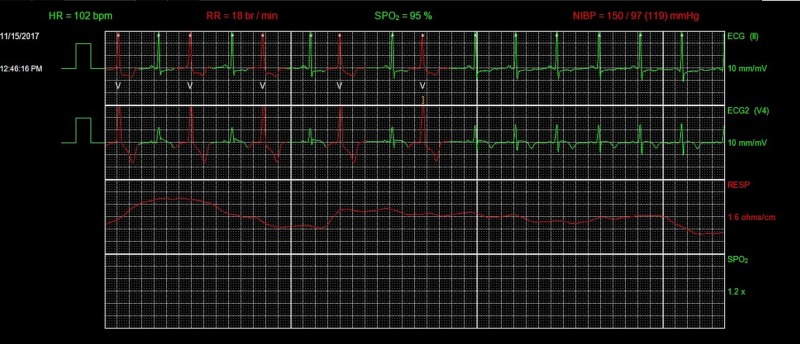
Telemetry showing resolution of AIVR. HR, heart rate; RR, respiratory rate; SPO_2_, peripheral capillary oxygen saturation; NIBP, non-invasive blood pressure; ECG, electrocardiogram.

The ECG on admission showed anterolateral ST-segment elevation (Figure 1). His vital signs at presentation included a heart rate of 112 beats per minute (bpm), blood pressure of 138/104 mmHg, respiratory rate of 14 breaths per minute, and oxygen saturation of 95% on room air. On physical examination, the patient appeared in no distress. First and second heart sounds were normal without murmur, rub, or gallop. Lungs were clear to auscultation. Serum chemistries were normal including potassium 3.8 mmol/L (3.5-5.1 mmol/L), bicarbonate 25 mmol/L (22-29 mmol/L), creatinine 0.9mg/dL (0.7-1.2 mg/dL), ionized calcium 1.12 mmol/L (1.12-1.3 mmol/L), ionized magnesium 0.45 mmol/L (0.43-0.61 mmol/L), and troponin T was undetectable at <0.01 ng/mL. Thyroid studies were unremarkable. Emergent coronary angiography was performed and showed no significant coronary artery disease. Repeat ECG in the cardiac catheterization lab showed resolution of ST-segment elevation with new T wave inversion in the anterolateral leads (Figure 2). The patient had a short run of ventricular tachycardia in the cardiac catheterization lab that resolved spontaneously. Transthoracic echocardiogram (TTE) was obtained and ruled out structural or valvular abnormalities and showed a normal ejection fraction. On hospital day two, the patient had an episode of chest pain, palpitation, and increased belching with associated telemetry findings of a wide complex rhythm (Figures 3-6) which was diagnosed initially as ventricular tachycardia with a rate of 101 bpm. Symptoms resolved with sublingual nitroglycerin. Further review of telemetry revealed ST-segment elevation in Lead V4 prior to the onset of the wide complex arrhythmia. This led to the final diagnosis of AIVR following an episode of Prinzmetal’s angina. Cardiac magnetic resonance imaging (MRI) was unremarkable for structural abnormalities. He was treated with diltiazem, isosorbide mononitrate, and nitroglycerin as needed. On further questioning, he mentioned that these episodes of chest pain had been going on for a few years and were initially attributed to his esophageal disease. Retrospectively, these episodes of chest pain were likely Prinzmetal’s angina.

## Discussion

Studies have found that the majority of arrhythmias associated with CAV were ventricular tachycardia, bigeminy, second and third degree atrioventricular (AV) block, and ventricular fibrillation [3-4]. AIVR has not been documented as an arrhythmia following CAV. Here we sought to present a case where our patient presented with typical CAV as evidenced by ST-segment elevation on ECG, unremarkable coronary angiography, with a normal TTE and cardiac MRI. During these episodes of CAV, the patient did not have troponin T elevation. A two-minute run of asymptomatic AIVR was documented following resolution of ST-segment elevation. The presence of AIVR brought our attention to its differential diagnosis, most commonly being reperfusion arrhythmia, which prompted us to look at telemetry prior to the event which revealed the diagnosis. A provocation test with ergonovine for a definitive diagnosis of CAV was not performed during cardiac catheterization although its use has been reported to induce coronary vasospasm [5].

Accelerated idioventricular rhythm is a slow (50-120 bpm) ventricular rhythm that consists of three or more consecutive monomorphic ventricular beats with gradual onset. It is usually a benign and self-terminating arrhythmia. It does not require specific anti-arrhythmic therapy unless in the presence of hemodynamic instability [6]. It has been used as a marker of successful reperfusion after thrombolytic therapy in ST-segment elevation myocardial infarction (STEMI) since 1950s [6-8]. It has also been described in healthy subjects, structural heart disease, lupus, pregnancy, spontaneous coronary artery dissection, hyperkalemia, dietary supplements such as hydroxycut gummies, as well as medications including digitalis and desflurane [6, 9-13]. Its clinical importance has been mainly in the prediction of successful thrombolysis after STEMI with a sensitivity of 45% and a specificity of 65% [7]. An abnormal calcium-dependent automatism has been hypothesized as a cause of AIVR in the presence of acute ischemia [14]. It can be hypothesized that this same mechanism causes AIVR in the setting of coronary vasospasm.

As part of the diagnostic approach to unexplained syncope, electrophysiological study (EPS) can be a useful tool. This was found to be most apparent in those subjects with concurrent structural heart disease [15]. There is currently no recommendation to perform an EPS for AIVR as this has historically been a marker of reperfusion rather than malignant arrythmia.

## Conclusions

Despite being an uncommon arrhythmia, the presence of AIVR after CAV should always be in the differentials to avoid unnecessary work up and treatment in the long run. Review of telemetry with correlation to symptomatic events can provide valuable insight into the etiology of arrhythmias and help in determining the correct diagnosis.
